# Parameter Estimation of a Ground Moving Target Using Image Sharpness Optimization

**DOI:** 10.3390/s16071017

**Published:** 2016-06-30

**Authors:** Jing Yu, Yaan Li

**Affiliations:** School of Marine Science and Technology, Northwestern Polytechnical University, Xi’an 710072, China; liyaan@nwpu.edu.cn

**Keywords:** moving target detection, SAR, parameter estimation, sharpness optimization

## Abstract

Motion parameter estimation of a ground moving target is an important issue in synthetic aperture radar ground moving target indication (SAR-GMTI) which has significant applications for civilian and military. The SAR image of a moving target may be displaced and defocused due to the radial and along-track velocity components, respectively. The sharpness cost function presents a measure of the degree of focus of the image. In this work, a new ground moving target parameter estimation algorithm based on the sharpness optimization criterion is proposed. The relationships between the quadratic phase errors and the target’s velocity components are derived. Using two-dimensional searching of the sharpness cost function, we can obtain the velocity components of the target and the focused target image simultaneously. The proposed moving target parameter estimation method and image sharpness metrics are analyzed in detail. Finally, numerical results illustrate the effective and superior velocity estimation performance of the proposed method when compared to existing algorithms.

## 1. Introduction

Ground moving target indication (GMTI) combined with synthetic aperture radar (SAR) has become a well-established technique for civil and military applications. The challenge of GMTI includes both the detection of targets and the estimation of their velocity components. Recently, the topic of moving target detection has been extensively studied [1,2,3,4,5]. In conventional SAR images, the target will be displaced and defocused because of the target’s radial (cross-track) and along-track velocities, respectively [6]. The target’s moving parameters, such as radial velocity, along-track velocity, and original azimuth position, play an important role in monitoring ground vehicles. Using these parameters, we can reposition them to the true azimuth location and extrapolate a target’s future position [7,8]. Generally, the interferometric phase is employed to determine the radial velocity while the along-track velocity is acquired by the target’s Doppler parameters. For a space-borne SAR system, the speed of ground slow moving targets are rather slow compared with the high speed of the satellite platform (typical platform velocities are in the range of 7000 m/s to 7500 m/s), therefore, the influence of the target’s along-track velocity can be ignored. In contrast, for airborne SAR systems, the target’s along-track velocity component will inevitably cause the image to be defocused, consequently reducing the detection capability.

In this paper, the main effort lays in the estimation of the motion parameters of a point moving target whose along-track velocity must be considered. Assuming that the moving targets have been detected using the displaced phase center antenna (DPCA) method, the along-track interferometric (ATI) method, or some other method, a new moving target parameter estimation algorithm based on the image sharpness optimization criterion is proposed. The target’s velocity components can be obtained by optimizing the sharpness cost function. The algorithm shows effective and superior velocity estimation performance in a number of simulations.

The paper is arranged as follows: Section 2 provides the echoes model of a typical SAR-GMTI system. In Section 3, we provide a brief introduction to the image sharpness metrics. In Section 4, the new moving target parameter estimation algorithm is described in detail. In Section 5, the algorithm is applied to the simulated SAR data; the performance of the algorithm is proved to be effective. In the closing section, a summary of the findings is presented.

## 2. Echoes Model of a Ground Moving Target

**Figure 1:**
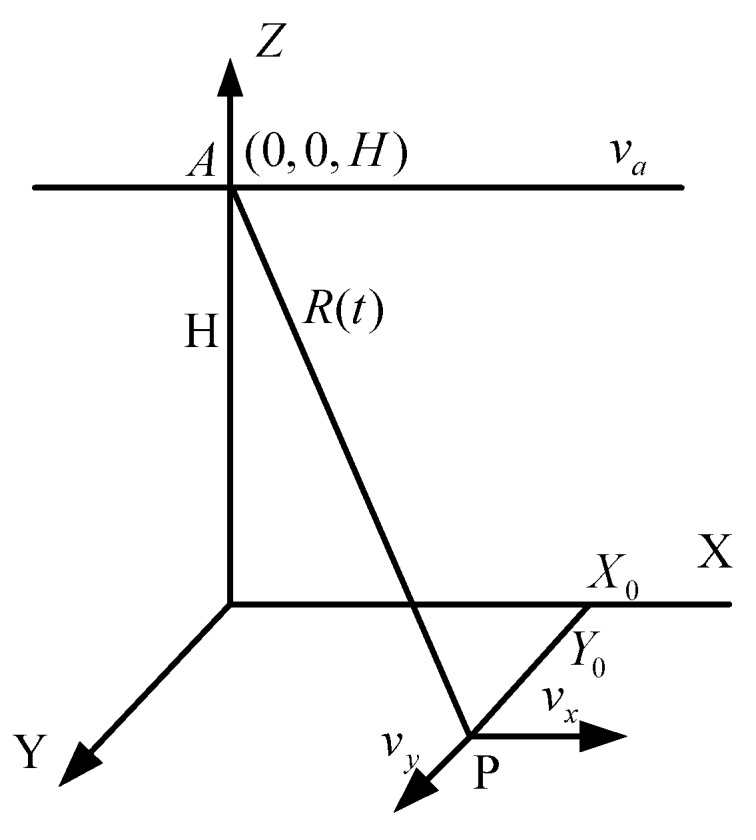
Geometry of a SAR system with a moving target.

Recently, the majority of SAR-GMTI algorithms utilize multiple apertures to provide an additional degree of freedom with which unwanted clutters may be suppressed. For simplicity and without loss of generality, we consider a single aperture SAR system model for the echoes backscattered from a ground point moving target [9,10]. Assume that the SAR system functions in side-looking mode with a fixed pointing angle orthogonal to the flight path. The moving target and radar geometry relation is illustrated in Figure 1, where the projection of the aircraft flying direction is defined as the *X*-axis, and the *Y*-axis is the radial (cross-track) direction, and the *Z*-axis represents the altitude. The platform moves at a constant speed *v_a_* and a fixed altitude *H*. An arbitrary point ground moving target *P* is assumed to be at position (*X*_0_,*Y*_0_,0) at *t* = 0 and moving with along-track and cross-track velocity components *v_x_* and *v_y_*, respectively. Suppose that when *t* = 0, the slant range of radar to target *P* is $R_{0}=\sqrt{H^{2}+X_{0}^{2}+Y_{0}^{2}}.$ For a small antenna azimuth beamwidth, it is possible to approximate the instantaneous range between radar and the point target by a second-order Taylor series:

(1) $$R(t)\approx R_{0}+\frac{v_{y}Y_{0}}{R_{0}}t-\frac{(v_{a}-v_{x})X_{0}}{R_{0}}t+\frac{v_{y}^{2}+{\left(v_{a}-v_{x}\right)}^{2}}{2R_{0}}t^{2}$$

Assuming the radar transmits a linear frequency modulated signal, after range compression, the signal can be expressed as:

(2) $$S_{1}(t)=A_{T}rect(\frac{t}{T})\exp(-j\frac{4\pi }{\lambda }R(t))$$

where *T* is the signal pulse period and *λ* is the wavelength.

After Fourier transformation, the signal can be expressed as follows:

(3) $$S_{1}(f)=A_{T}\sqrt{\frac{1}{\left|f_{dr}\right|}}rect\left(\frac{f+f_{dc}}{B}\right)\exp\left(-\frac{j2\pi f_{dc}f}{f_{dr}}\right)\exp\left(\frac{-j\pi f^{2}}{f_{dr}}\right)$$

where *A_T_* is the magnitude, *B* is the transmission bandwidth, *f_dc_* is the Doppler center frequency of the target, and *f_dr_* is the Doppler rate of the target. Derived from Equation (1), *f_dc_* and *f_dr_* can be given as:

(4) $$f_{dc}=-\frac{2}{\lambda }\times \frac{dR(t)}{dt}=\frac{2}{\lambda R_{0}}\left[X_{0}(v_{a}-v_{x})-v_{y}Y_{0}\right]$$

(5) $$f_{dr}=-\frac{2}{\lambda }\times \frac{d^{2}R(t)}{dt^{2}}=-\frac{2}{\lambda R_{0}}\left[{\left(v_{a}-v_{x}\right)}^{2}+v_{y}^{2}\right]$$

For a stationary scene, the Doppler center frequency and Doppler rate are:

(6) $$f_{dc0}=f_{dc}|{}_{v_{x}=0,v_{y}=0}=\frac{2X_{0}v_{a}}{\lambda R_{0}}$$

(7) $$f_{dr0}=f_{dr}|{}_{v_{x}=0,v_{y}=0}=-\frac{2v_{a}^{2}}{\lambda R_{0}}$$

In SAR imaging, the reference function used in azimuth processing is constructed using the stationary target’s Doppler parameters. The reference function can be formed as follows:

(8) $$sref_{a}(f)=\exp(j\pi f^{2}/f_{dr0})$$

Synthetic aperture radar can image a stationary scene with fine resolution. However, for a moving target, smearing, defocusing, and displacement are inevitable because the Doppler parameters of a moving target are different from those of a stationary scene.

Consequently, the signal after azimuth processing in the Doppler domain can be expressed as:

(9) $$S_{1}^{\prime }(f)=A_{T}\sqrt{\frac{1}{\left|f_{dr}\right|}}rect\left(\frac{f+f_{dc}}{B}\right)\exp\left(-\frac{j2\pi f_{dc}f}{f_{dr}}\right)\exp\left(-\frac{j\pi f^{2}}{f_{dr}}+\frac{j\pi f^{2}}{f_{dr0}}\right)$$

From Equation (9), for a moving target, the quadratic phase term is not equal to zero because *f_dr_* ≠ *f_dr_*_0_. In other words, the Doppler rate of the moving target is not completely compensated and there still exists quadratic phase errors. For SAR images, the linear phase errors may cause image displacement, whereas the quadratic phase errors may introduce image defocus and reduce the signal amplitude. If the quadratic phase errors can be estimated and fully compensated for the signal, then the image will be focused and the signal energy will be converged in the center, see Equation (11).

Let *γ* represent the quadratic phase error in Equation (9):

(10) $$\begin{array}{cc} \gamma & =\frac{j\pi }{f_{dr}}-\frac{j\pi }{f_{dr0}}\\ & =j\pi \left\{\frac{\lambda R_{0}}{2X_{0}v_{a}}-\frac{\lambda R_{0}}{2\left[{\left(v_{a}-v_{x}\right)}^{2}+v_{y}^{2}\right]}\right\} \end{array}$$

After phase error compensation, the focused image in the Doppler domain can be expressed as:

(11) $$S_{compen}^{\prime }(f)=S_{1}^{\prime }(f)\exp(\gamma f^{2})$$

After compensation, the quadratic phase is equal to zero, see Equation (12):

(12) $$-\frac{j\pi f^{2}}{f_{dr}}+\frac{j\pi f^{2}}{f_{dr0}}+\gamma =0$$

One of the measurements of the degree of image focus is named image sharpness. In the next section, we will provide a brief introduction to image sharpness metrics.

## 3. Image Sharpness Metrics

Sharpness is considered as the ratio of the difference in luminance of an object and its immediate surroundings. In SAR images, sharpness can be considered as a measurement of the degree of the focus of the image because the sharpness allows one to emphasize the difference in the intensity of the scene. The amplitude of the focused image is concentrated in the center pixels. As a result, the focused image has several pronounced peaks corresponding to the scatters of the target that produce high fluctuations of the image intensity. Conversely, the amplitude of the unfocused image exhibits small fluctuations around its mean value. Thus, we expect a high sharpness value of the focused image because there are great differences in the intensity. Instead, the amplitude of an unfocused image is concentrated around its mean value and the sharpness value is low.

Because of unknown platform movement and ground target motion, SAR suffers from image degradation due to the presence of phase errors in the received signal. These phase errors act as a blurring filter, resulting in loss of resolution, spurious targets and a decrease in image sharpness. If the quadratic phase errors can be estimated and compensated for the signal, then the image can be focused and the sharpness value will reach the highest value. Determining how to estimate the phase errors is the key problem for image focusing.

**Figure 2:**
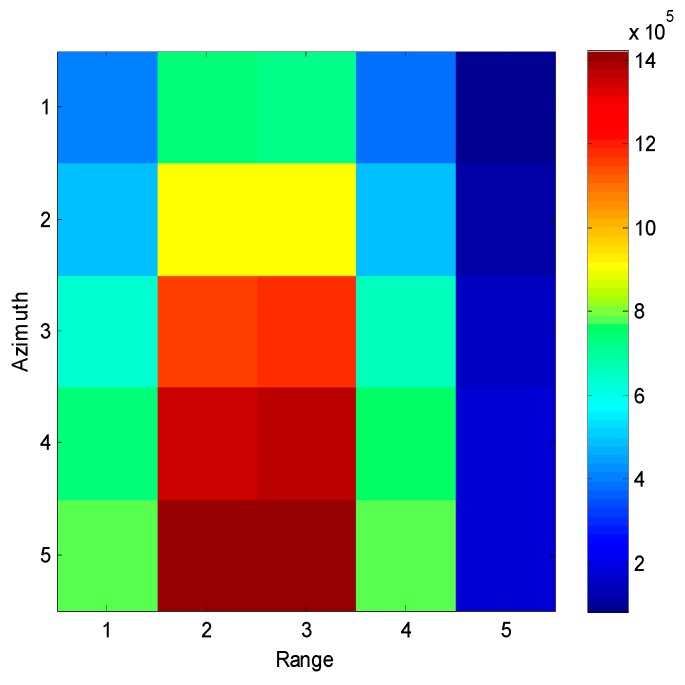
A part of a SAR image.

Figure 2 shows a small part of real SAR image; it contains 5 × 5 pixels. The amplitude of each pixel is presented by different colors.

Let *I_i,j_*(*γ*) represent the complex value of pixel (*i,j*) in the phase compensated image $S_{compen}^{\prime },$ which is a function of *γ*.

Image sharpness is used to evaluate the degree of focus [11,12,13,14,15]. Because of the point-like nature of the SAR image model, maximizing sharpness is found to increase the image focus. Because the complex value of the compensated image pixel is a function of *γ*, the sharpness can also be expressed as a function of *γ*.

In this paper, we study the standard deviation intensity sharpness function, as proposed in [9]:

(13) $$C(\gamma )=\frac{\sqrt{A\left\{{\left[\sum_{i=1}^{M}\sum_{j=1}^{N}I_{ij}^{2}(\gamma )-A\left\{\sum_{i=1}^{M}\sum_{j=1}^{N}I_{ij}^{2}(\gamma )\right\}\right]}^{2}\right\}}}{A\left\{\sum_{i=1}^{M}\sum_{j=1}^{N}I_{ij}^{2}(\gamma )\right\}}$$

where *M* and *N* are the number of pixels in range and azimuth directions, respectively. *A*{•} is the spatial mean operator. As an example, application of *A*{•} to a real sequence *x*(*p*) with *p* = 1,…,*p* is shown in Equation (14):

(14) $$A[x(p)]=\frac{1}{p}\sum_{p=1}^{P}x(p)$$

Sharpness optimization is a method by which a quadratic phase error estimate is chosen to maximize a particular cost function, see Equation (15):

(15) $$\overset{⏜}{\gamma }=\underset{\gamma }{\arg\max}C(\gamma )$$

The properties of the sharpness function *C*(*γ*) have been analyzed in detail in [15]. When *C*(*γ*) achieves the maximum value, the quadratic phase error is completely compensated and the image will be focused. The variable *γ* corresponds to the estimated quadratic phase errors, which is presented by $\overset{⏜}{\gamma }$.

**Figure 3:**
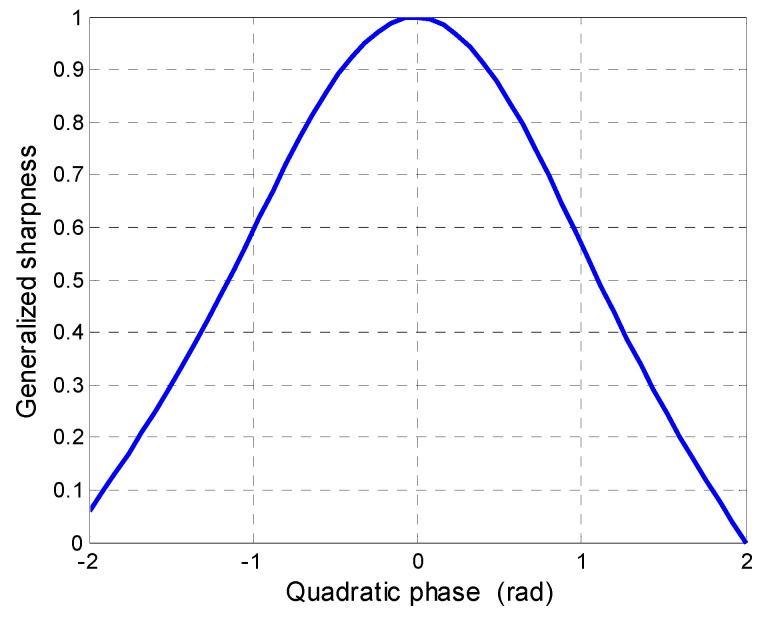
Relationship between the sharpness value and the quadratic phase.

For example, Figure 3 illustrates the relationship between the sharpness value and the quadratic phase. For a point moving target, when the quadratic phase error is fully compensated, the quadratic phase coefficient is equal to zero, and the sharpness value is maximized.

In SAR imaging, sharpness metrics are always used for image autofocus. Through the process of estimating the phase errors and compensating them for the unfocused image, a higher definition image can be acquired. For a moving target, the velocities introduce a phase error, thus causing the image to be unfocused. The phase error of a moving target can also be estimated using sharpness optimization metrics. Thus, inspired by the concept of sharpness, we proposed a moving target parameter estimate method in the next section.

## 4. Parameter Estimation Using Sharpness Optimization

### 4.1. Coarse Parameter Estimation

Traditionally, after clutter suppression and moving target detection in the image domain, the motion parameter of the target can be estimated using ATI [16,17] and DPCA [18,19] techniques.

For ATI techniques, the velocities of the ground moving target can be obtained via the interferometric phase, using Equations (16) and (17):

(16) $${\overset{⏜}{X}}_{0}=\frac{\lambda R_{0}\overset{⏜}{\phi }(\overset{⏜}{f})}{2\pi d}+\frac{d}{4}$$

(17) $${\overset{⏜}{v}}_{y}=v_{a}\frac{{\overset{⏜}{X}}_{0}}{Y_{0}}-\frac{\lambda R_{0}\overset{⏜}{f}}{2Y_{0}}$$

In which $\overset{⏜}{\phi }\left(f\right)$ is the interferometric phase, *d* is the distance between phase centers, and the other parameters are the same as mentioned before.

Because the image of moving target is unfocused, the interferometric phase $\overset{⏜}{\phi }\left(f\right)$ is also inaccurate. The motion parameters obtained by Equations (16) and (17) may have large errors. To improve the estimation accuracy, we propose an accurate target motion parameter estimation method that estimates the parameters and obtains the focused image simultaneously.

### 4.2. Accurate Parameter Estimation

Derived from Equation (10), the quadratic phase error *γ* is a function of the along-track velocity *v_x_* and the radial velocity *v_y_*. From Figure 3, if the quadratic phase error is completely compensated, then the sharpness function reaches peak amplitude and the image is fully focused. Thus, the estimation of the values of *v_x_* and *v_y_* involve an optimization problem of the function of Equation (15).

The properties of the sharpness function *C*(*γ*) have been analyzed in detail in [13]. *C*(*γ*) has been proven to be a unimodal function, which has only one maximum value. The maximum value corresponds to a focused image.

The unknown variables in *C*(*γ*) are *v_x_* and *v_y_*, which are independent variables. The problem of Equation (15) can be simplified as two independent one-dimensional unimodal function optimization problems. Searching *v_x_* and *v_y_*, when *γ* equals to the real quadratic phase errors, *C*(*γ*) reaches the peak value. The corresponding *v_x_* and *v_y_* are the true velocity components of the target.

From Equations (16) and (17), a coarse estimation of *v_y_* can be acquired. As a result, the searching intervals of *v_y_* can be set centered on ${\overset{⏜}{v}}_{y}$, which can substantially reduce the computational load. The searching intervals of ${\overset{⏜}{v}}_{x}$ can be set adequately large. As the searching process is constrained to a small image area containing the moving target, the algorithm is not time consuming.

**Figure 4:**
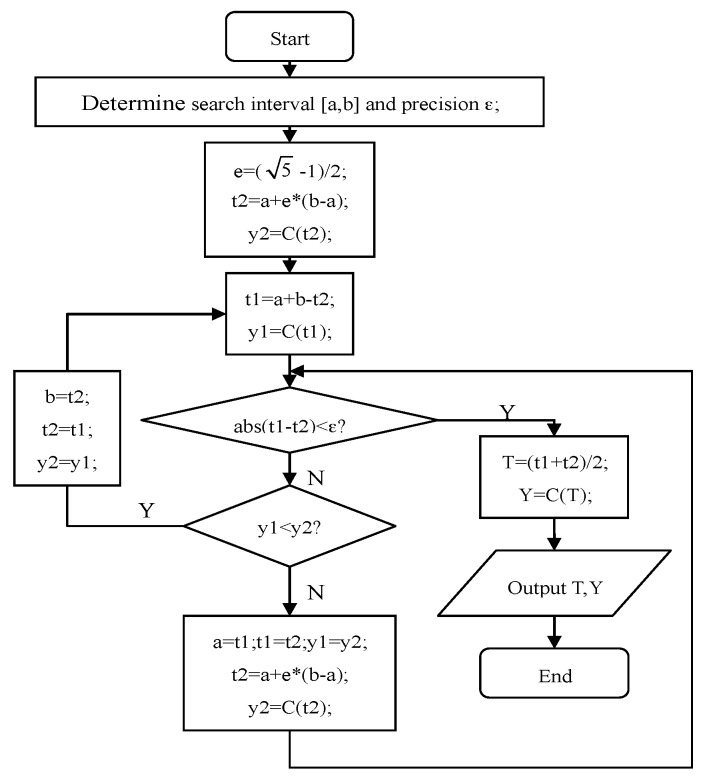
Flowchart of the golden-section search method.

Because *C*(*γ*) contains only one maximum, unimodal function optimization methods can be applied to effectively solve the optimization problem, such as the advance-and-retreat search method [20] and the golden-section search method [21]. Taking as an example the golden-section search method, whose flowchart is illustrated in Figure 4.

The searching steps can be summarized below:

(1) Choose the search interval of *v_y_*: [*v_ya_*,*v_yb_*].
(2) Choose the search interval of *v_x_*: [*v_xa_*,*v_xb_*].
(3) Let *v_y_* = *v_ya_*; search *v_x_* in [*v_xa_*,*v_xb_*], set *ε* = 0.001. According to the process shown in Figure 4, obtain the maximum value $C_{a}^{*}$ and the corresponding $v_{ax}^{*}$.
(4) Let *v_y_* = *v_yi_* = *v_ya_* + Δ*y*, where Δ*y* is the step size acquired by the flowchart in Figure 4. According to the process shown in Figure 4, obtain the maximum value $C_{i}^{*}$ and the corresponding $v_{ix}^{*}$.
(5) Repeat Step (4); until *v_y_* = *v_yb_*, and then obtain $C_{b}^{*}$ and $v_{bx}^{*}$.
(6) Let $C_{\text{max}}^{*}=\text{max}\{C_{a}^{*},\ldots ,C_{b}^{*},$ the corresponding *v_x_* and *v_y_* are the estimates of the target’s velocity components.

### 4.3. Flow Chart of Parameter Estimation of a Ground Moving Target

**Figure 5:**
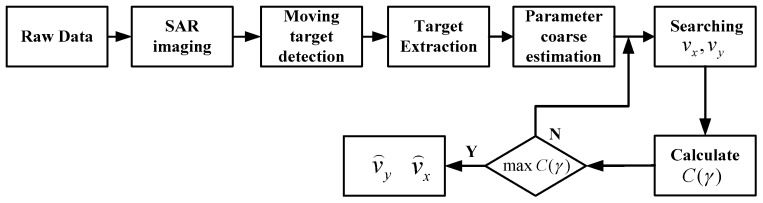
Flowchart of the moving target parameter estimation process.

Based on the principle presented above, the flowchart of the proposed approach is illustrated in Figure 5. The main steps of the ground moving target parameter estimation algorithm using sharpness optimization are summarized as follows:

(1) Imaging of the raw SAR data.
(2) Clutter suppression and detection of the moving target in the image domain.
(3) Extraction of the small part of the image containing the moving target.
(4) Corse estimation of the motion parameters.
(5) Joint searching of *v_x_* and *v_y_* to obtain the quadratic phase error.
(6) Calculation of the sharpness cost function *C*(*γ*).
(7) Repeat Steps (5) and (6); when *C*(*γ*) achieves the maximum value, stop. The corresponding *v_x_* and *v_y_* are the accurate along-track and radial velocity of the target, respectively.

## 5. Experimental Results

**Figure 6:**
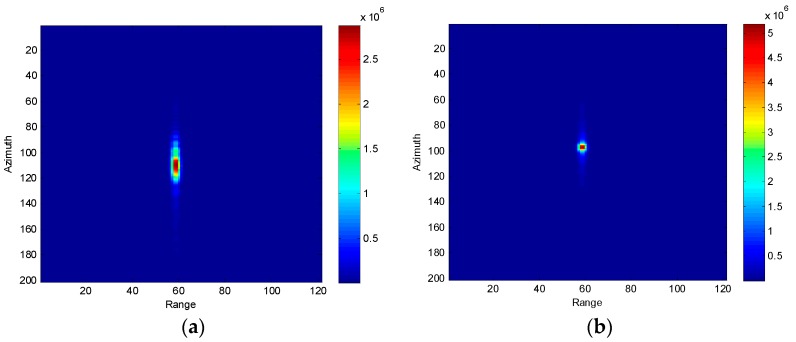
(**a**) Unfocused image of the moving target; (**b**) Focused image of the moving target.

In this section, we present and discuss a number of results obtained by applying the proposed moving target parameter estimation method to simulated SAR data. The simulation conditions are: aircraft velocity = 150 m/s, transmission wavelength = 0.03 m, pulse repetition frequency = 500 Hz, and distance between phase centers = 0.96 m. The parameters of the moving target are: radial velocity = 2 m/s, along-track velocity = 4.5 m/s and original azimuth position = 130 m. Following the procedure shown in Figure 5, following imaging, detection, and extraction, the image of the moving target is depicted in Figure 6a. 

**Figure 7:**
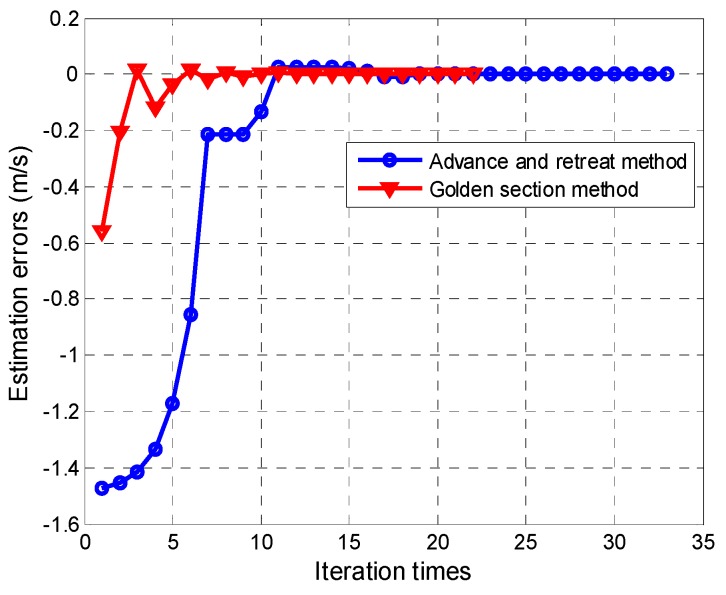
Estimation errors of the quadratic phase coefficient versus the iteration times.

**Figure 8:**
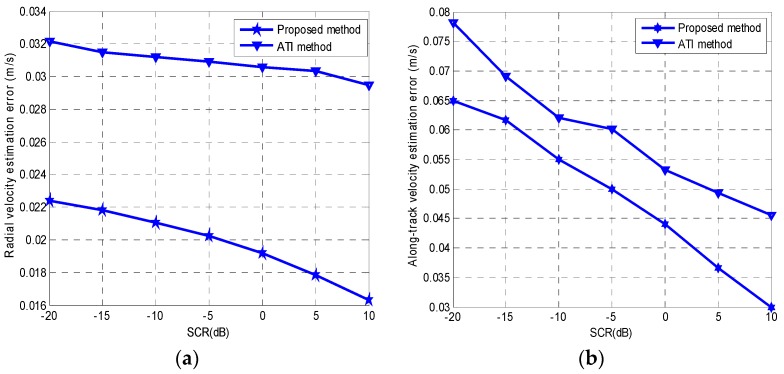
(**a**) Radial velocity estimation error versus the SCR; (**b**) Along-track velocity estimation error versus the SCR.

Because of the along-track velocity, the image is unfocused and spread along the azimuth direction. Both the advance-and-retreat search method and the golden-section search method are applied to solve the optimization problem. The iteration process is shown in Figure 7. From Figure 7, we can infer that the golden-section search method has a superior convergence rate than the advance-and-retreat method. For the moving target in Figure 6a, under the same computer conditions, the golden-section search method takes 1.292 s, whereas the advance-and-retreat search method takes 1.873 s to obtain the optimal estimation of the target velocities. The results show that the moving target parameter estimation proposed in this paper has a very low computation load. The estimated moving target parameters are: along-track velocity = 4.503 m/s, radial velocity = 2.032 m/s and original azimuth location = 124.39 m. The estimated parameters are in agreement with the simulated parameters. Figure 6b shows the focused image obtained by compensating the quadratic phase errors. We also compared the performance of the proposed parameter estimation method with the traditional ATI method. Figure 8a shows the radial velocity estimation error versus the signal-to-clutter ratio (SCR), and Figure 8b shows the along-track velocity estimation error versus the SCR. The proposed method is found to exhibit higher velocity estimation accuracy than the traditional ATI method.

## 6. Conclusions

This paper introduced a novel motion parameter estimation method using contrast optimization for a ground moving target. The echo model of a target in a SAR image was analyzed and the relationships between the quadratic phase errors and the target’s velocity components are derived first. Next, the concepts of image sharpness metrics were discussed in detail. Based on the sharpness optimization criterion, the peak magnitude of the sharpness cost function corresponds to the real phase errors, which are functions of the target’s velocity components. Using two-dimensional searching, we can obtain the along-track and radial velocities of the target. The proposed method can obtain the motion parameters and the focused image simultaneously, and obviously enhance the estimation accuracy. In the simulation section, the method was applied to simulated SAR data, and the results illustrated that the proposed method can effectively estimate the moving target parameters with better accuracy than the conventional ATI method.
